# Monitoring the Impact of Artificial Structure on Hydrogeological Environment: A Case Study of Hydraulic Tunnel at Pirot Hydropower Plant

**DOI:** 10.3390/s24206578

**Published:** 2024-10-12

**Authors:** Marina Čokorilo Ilić, Miroslav P. Popović, Dragoljub Bajić, Vesna Matović, Filip Abramović, Filip Alimpić

**Affiliations:** 1 Environment and Sustainable Development Program, Singidunum University, Danijelova 32, 11010 Belgrade, Serbia; marinacokorilo@googlemail.com (M.Č.I.);; 2 Faculty of Mining and Geology, University of Belgrade, Đušina 7, 11120 Belgrade, Serbia; 3 Ministry of Environmental Protection, Government of Serbia, Omladinskih Brigada 1, 11070 Belgrade, Serbia

**Keywords:** hydropower tunnel, groundwater, hydrogeological monitoring, piezometers, physicochemical sensing, hydrometry, cross-correlation

## Abstract

Artificial objects, particularly tunnels used for water transport under pressure, impact the geological and hydrogeological environment to a greater or lesser extent, and it is vital to assess their contributions to groundwater quality. Although tunnels are typically lined with concrete, their interaction with the hydrogeological environment intensifies over time. In this study, the detailed spatiotemporal monitoring of all hydrogeological features within the potential influence zone of the hydraulic tunnel of the Pirot Hydropower Plant has been conducted in order to determine the degree of interaction between the artificial object and the natural environment in real time, and to assess the correlation between monitored parameters. Natural conditions of the environment were defined, as well as potential changes through the observing groundwater regimes. The monitoring network included observations of groundwater regimes at seven springs located in close proximity to the hydraulic tunnel, within the tunnel, at three piezometers, and along the river, while methods employed were hydrological monitoring, physicochemical monitoring, and groundwater piezometer sensing. Cross-correlation analysis has been applied for assessing the impact of precipitation dynamics on the spring discharge regime. The results indicate a direct influence of the tunnel on the hydrogeological environment, proving the consistency and high correlation between the monitored parameters.

## 1. Introduction

The hydrogeological system represents a system defined by specific input elements, environmental characteristics, processes within the observed environment, and output elements, with input and output elements being interdependent [1]. The definition of the hydrogeological system itself indicates the complexity of the processes that occur within it, making its understanding and study in most cases very demanding and requiring a thorough examination of all components. The natural hydrogeological system is defined by its input and output parameters, i.e., natural recharge and discharge zones. This implies a natural process of groundwater circulation, based on the hydrogeological water balance [2]. Groundwater circulation within the hydrogeological system occurs within different types of hydrogeological structures where various conditions of their distribution exist. Two types of hydrogeological systems are distinguished: systems formed in basin structures and systems formed in deep fault structures [3,4,5,6].

In addition to local (shallow) systems, complex hydrogeological systems also include intermediate (transitional) and regional (deep) hydrogeological systems. In basin structures, local hydrogeological systems have recharge and discharge zones that are directly influenced by atmospheric conditions, which directly affect the physicochemical characteristics of groundwater. In regional systems, recharge and discharge zones are completely independent, and groundwater in these systems has a stable regime due to the longest residence time in the geological environment [7]. Interaction between local and regional hydrogeological systems usually occurs in the intermediate (transitional) system, where there is often mixing of groundwater with different physicochemical characteristics [8].

Due to the circulation process and the different residence times of groundwater in the hydrogeological environment, there is a change in their chemical composition [4,9]. Hydrogeological systems formed in deep/regional fault structures are characterized by a much more complex process of groundwater circulation compared to circulation occurring in basin structures because conduits are formed in various metamorphic and igneous rocks, carbonate, and terrigenous formations [6,10].

When such a complex natural hydrogeological system is intersected by an artificial structure, such as a hydraulic tunnel used for pressurized water transport, despite grouting operations and lining of the structure, interaction between transported water and natural groundwater can occur. The water transported under high pressure through the artificial structure can cause numerous changes upon entering the natural hydrogeological environment, primarily in terms of disrupting natural groundwater circulation patterns and mixing waters from different systems characterized by different physicochemical properties. As carbonate rocks indicate a predominant development of karst systems, groundwater circulation from different hydrogeological systems can negatively impact the bedrock (dissolution), thereby compromising its stability. The spring discharge regime is greatly affected by the precipitation regime, especially in karst areas. Artificial interventions in karst terrains can cause various ecological problems, such as changes in the quality and quantity of water, groundwater and surface flow regimes [11,12,13,14,15,16,17,18], not only during their construction but also later, during the exploitation process. The impact of tunnel construction on karst groundwater systems may lead to a decrease in the water level of karst springs [19], a concentrated channel of mud and water gushing [20,21] or decreasing of soil moisture content [22]. In addition to that, tunnel construction may also cause alterations in the groundwater chemistry and later interaction of transported water and groundwater in hydrogeological systems, which require hydro-geochemical analyses using complex multivariate statistical techniques [23,24,25,26,27]. Therefore, it is necessary to assess the disturbance in a hydrogeological system caused by tunnel construction and to evaluate the ability of a groundwater system to recover [18]. A number of studies have been performed on these investigations [10,26,27,28,29,30,31,32,33,34], with most of them being evaluated by statistical or numerical analysis methods [24,28,30,32,33]. In order to monitor groundwater areas with artificial objects regionally and locally and to address potential risks, on-site methods including piezometer measurements and electrical conductometry enable the determination of parameters such as the degree of interaction between groundwater and water in the tunnel, and physicochemical characteristics of the groundwater [10,35,36]. Numerous water balance models have been used in order to quantify the hydrogeological impact of tunnel construction on spring discharge and hydrographs of the flow structure of karst aquifers [28,29,30,31,32,33]. However, a lack of long-term monitoring data and hydrological studies, particularly in the Balkans karst region (southeast Europe), raises the interest in these topics in the scientific and engineering community.

In this study, we employ various physical and chemical sensors for monitoring the impact of the tunnel onto the groundwater and environment, in order to assess these impacts and to evaluate the efficiency and mutual consistency of these methods. The zone of the hydraulic tunnel of the Pirot Hydropower Plant (HPP Pirot) and the zone of potential impact of this structure on the natural hydrogeological system have been analyzed as the research area. Based on successive hydrological measurements, the impact of different tunnel operation regimes on the flow of the Dobrodolska River, as well as on the springs located in the zone of potential influence of the tunnel, has been assessed. The influence of the degree of interaction between groundwater and water transported by the tunnel under pressure on spatial distribution of groundwater drawdown has been considered in detail, and the potential impact on the natural hydrogeological environment has been also discussed. Besides the quantitative characteristics of groundwater, physical and chemical parameters of groundwater were considered over the observation period as being crucial for understanding the natural process of groundwater circulation and for assessing the impact of the tunnel on water exchange. The analysis of the impact of these two variables has been performed based on the cross-correlation analysis for the entire observation period.

## 2. Materials and Methods

### 2.1. Study Area

**Figure 1:**
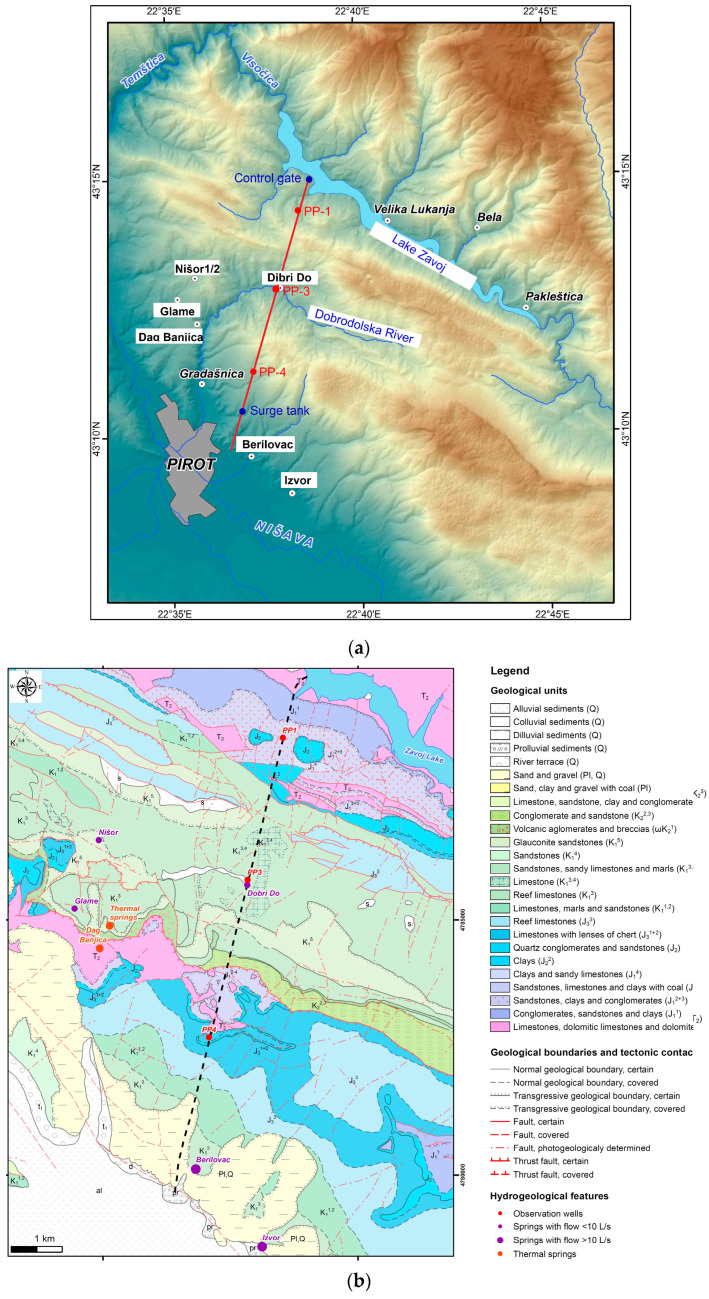
(**a**) Location of the research area and the route of Pirot Hydropower Plant (HPP Pirot) tunnel with its accompanying structures (7 springs); (**b**) simplified geological map of the study area based on the basic geological map of Yugoslavia [10,37]. Dashed line denotes the water-conveyance tunnel.

The research area is located in southeastern Serbia, in the central part of the Stara Planina mountain range (Figure 1a) near the city of Pirot (the largest city of this region), situated within the geographical coordinates of 43°08′–43°15′ N latitude and 22°35′–22°38.5′ E longitude, at an elevation between 370 and 910 m above sea level. It covers an area of roughly 160 sq. Km, and is located near Lake Zavoj and Visočica River. The climate is sub-mountainous, as indicated by the processed data on air temperatures and precipitation from Dimitrovgrad meteorological station (over the period from 1990 to 2016). The area experiences winters from November through March, with temperatures ranging from 0 °C to 3 °C, while the summer season is shorter and lasts from June through September, and having an annual mean temperature 18 °C–38.8 °C. Total length of the water conduction tunnel (Pirot Hydropower Plant—HPP tunel) is 9 km and it connects water reservoir “Zavoj” to the electricity generator turbines. Water is transported through the tunnel under maximum pressure, approximately 8 bars. The site of this study consists of HPP tunnel, seven springs, and Dobrodolska River that, in one of its courses, passes through the discharge zone of the fractured thermal spring Dag Banjica.

#### Geological Conditions

In geological terms, the tunnel was constructed through the contact of two regionally significant tectonic units (Getic and Danubian), represented by a system of east-vergent thrusts [27]. The study area mainly consists of carbonate sediments from the Triassic, Jurassic, and Cretaceous periods (Figure 1b). Triassic deposits include limestones, dolomites, conglomerates, and sandstones, while Jurassic deposits consist of sandstones, clays, conglomerates, and marble limestones. The most common Cretaceous deposits are limestones, marls, and sandstones. The Mesozoic complex is overlain transgressively by Pliocene sands and marly clays [10].

### 2.2. Methods

#### 2.2.1. Groundwater Piezometry

Defining the natural regime of groundwater was conducted at 7 springs (Berilovac, Izvor, Nišor 1, Nišor 2, Glame, Dobri Do, Dag Banjica), along the Dobrodolska River, by three conventional piezometers (measuring groundwater pressure) located along the tunnel route and inside the hydropower tunnel itself during the period when the facility was out of operation (workover period). Water pressure is being converted into a frequency signal as the change in pressure on piezometer’s diaphragm causes change in steel wire tension. The vibration of the wire in the proximity of the magnetic coil generates a frequency signal that is transmitted to the readout device [38]. The research lasted a total of 108 days and covered a three-month period (September, October, and November 2016), during which data on quantitative and qualitative characteristics of groundwater were collected during different tunnel operation regimes (Table 1). Phase A was a 23-day period of tunnel being in operation (pressurized tunnel), followed by phase B (tunnel out of operation for 35 days), while phase C comprised a 50-day observation period at springs, and the extended period of 83 days involved observations at piezometers.

#### 2.2.2. Meteorological Data Acquisition

Meteorological monitoring has been used to define the climatic characteristics of the study area, primarily the temperature and precipitation regime, with the aim of understanding their impact on the natural hydrogeological regime. Daily precipitation and temperature data for the research area were obtained from the meteorological yearbooks of the Republic Hydrometeorological Service from the Dimitrovgrad meteorological station, measured using standard methodological procedures and instruments (rain gauge and pluviograph).

#### 2.2.3. Hydrological and Hydrogeological Monitoring

Hydrological research methods were carried out to determine the dependence between groundwater and surface water on water levels and stream flow at designated water measuring stations. Water level observations at established measurement stations were conducted using installed staff gauges, while stream flow measurements were carried out using an EasyFlow (Madd Technologies, Yverdon-les-Bains, Switzerland) instrument in various hydrological conditions to establish flow curves. The measurement procedure included the injection of a known mass of tracer (NaCl diluted in a certain amount of river water) into river, while the probe situated downstream measures the electrical conductivity of water flow carrying the dissolved salt. Based on linear dependence of electrical conductivity on salt concentration, and concentration being a function of time, the flow rate is simply calculated by integrating the concentration over time as follows:

$$Q=\frac{M}{\int_{0}^{T}\left(C_{t}-C_{0}\right)\;dt\;}$$

where *Q* is flowing rate (L/s), *M* is the mass of injected tracer (mg), *C_t_* is the actual salt concentration in water (mg/L) at the moment t, *C*_0_ is the initial concentration (mg/L), and *T* is total duration of measurement (s). Technical details of the EasyFlow instrument are given in Table 2.

**Figure 2:**
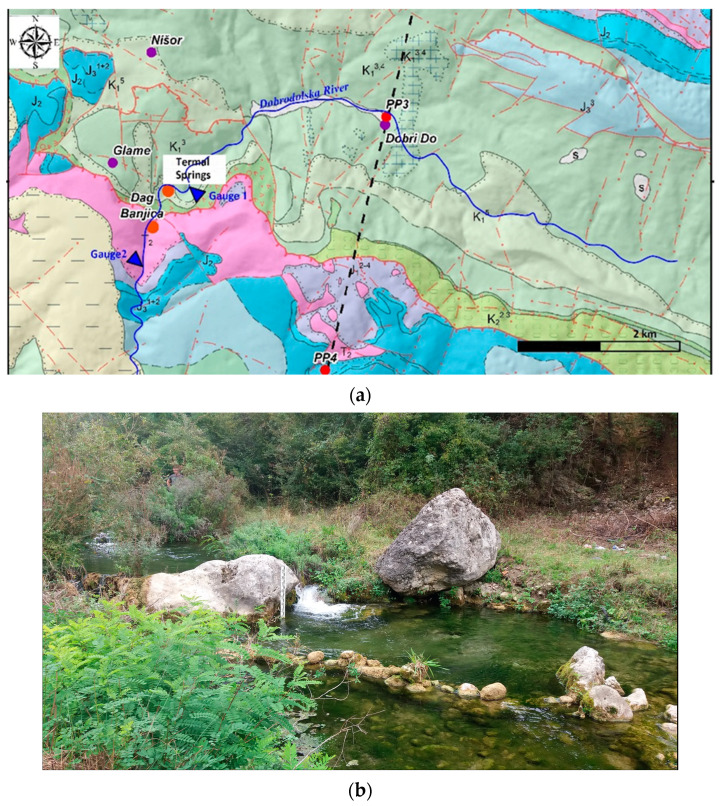
(**a**) Dobrodolska River showing the positions of staff gauge stations and geological terrain structure [10,37], and (**b**) a photograph of gauge 2 on the Dobrodolska River.

The measurements were conducted at two staff gauge stations: upstream (gauge 1, at an elevation of 471 m above sea level) at the point where the discharge zone of the thermal springs begins, and downstream (gauge 2, at an elevation of 432 m above sea level) where the discharge of thermal waters ceases (Figure 2). Within this zone, the largest thermal spring—Dag Banjica, which is primitively tapped, is located. The standard daily water level monitoring, accompanied by water discharge measurement on a seven-days basis (i.e., once in seven days) has been applied, providing the relevant flow rate function as the output.

Hydrogeological methods were applied to define the quantitative and qualitative characteristics of groundwater across the entire study area. These methods were crucial for determining the types of hydrogeological systems, assessing the degree of interaction between groundwater and water transported by the tunnel under pressure, and assessing the potential impact of the artificial structure on the natural hydrogeological environment. Observations of quantitative characteristics at all springs located in the potential impact zone of the hydropower tunnel were conducted using volumetric methods or hydrological measurements. In this method, water flow rate, *Q*, is determined by the following formula:

$$Q=\frac{V}{t}$$

where *V* is the volume of the vessel where water is being accumulated, and *t* is time period needed for empty vessel to fill.

Frequent monitoring of the observation wells along the tunnel was performed using Eijkelkamp TD-divers for monitoring quantitative characteristics (water pressure and temperature) of groundwater in piezometers, with a 2 min sampling interval [10]. The diver consists of a water pressure sensor, a temperature sensor, a memory storage unit, and a battery. It is a data-logger, i.e., it samples data in its memory unit over sampling time interval, defined by user.

#### 2.2.4. Physicochemical Monitoring

The observations of basic physicochemical characteristics of groundwater (pH value, total dissolved solids (TDS), temperature (T), electrical conductivity (EC), oxidation–reduction potential (ORP), dissolved oxygen (DO)) were carried out using a portable multiparameter probe HI9298194 (of HANNA manufacturer, Woonsocket, RI, USA) in situ throughout the entire observation period, at all springs. This probe is equipped with 20 m long cable, and possesses maximum logging capacity of 45,000 data, with logging time interval ranging from 1 s up to 3 h. Technical details of the instrument are listed in Table 3.

The primary chemical composition of groundwater (HCO_3_^−^, Cl^−^, SO_4_^2−^, Na^+^, K^+^, Ca^2+^, Mg^2+^) was determined using standard analytical instrumental lab methods (listed in Table 4).

#### 2.2.5. Cross-Correlation

Cross-correlation analysis is used to assess time-dependent random variables (in this case spring discharge and precipitation). In cross-correlation analyses, the correlation between a time-dependent variable and an independent variable can be quantified by computing cross-correlation coefficients at different time steps [36,39]. For correlation analysis in this study, the daily monitoring of water level (H) and a weekly measurement (once in a week) of discharge (Q) has been performed on gauges 1 and 2. From these measurements, the flow curve (Q = f(H)) has been derived, and from the curve the particular daily values, needed for cross-correlation analysis, have been read out.

The cross-correlation coefficient for any time step *k* is derived from the following equation [39]:

$$r_{k}=\frac{COV(x_{i},\;y_{i+k})}{\sqrt{VAR(x_{i}})\cdot VAR(y_{i})}$$

- *COV* is the covariance between two time-series
- *x_i_* is the independent variable (daily precipitation total time-series)
- *y_i_* is the the dependent variable (daily average discharge time-series),
- *VAR* (*x_i_*) and *VAR* (*y_i_*) are the covariances of the time-series of the two variables.

The covariance is obtained from the following equation:

$$COV\left(x_{i},y_{i+k}\right)=\frac{1}{n-k}\sum_{i=1}^{n-k}(x_{i}-\bar{x})\cdot (y_{i+k}-{\bar{y}}_{i+k})$$

and the variance from the time-series of the variables from the following:

$$VAR\left(x_{i}\right)=\frac{1}{n-k}\sum_{i=1}^{n-k}({x_{i}-\bar{x})}^{2}$$

$$VAR\left(y_{i}\right)=\frac{1}{n-k}\sum_{i=1}^{n-k}({y_{i+k}-{\bar{y}}_{i+k})}^{2}$$

Cross-correlation analysis was conducted on 5 springs (Izvor, Berilovac, Nišor 2, Dobri Do, Dag Banjica) and two observation sites on the Dobrodolska River (gauges 1 and 2).

The accuracy of the monitored results is ensured based on highly reliable instruments which have been calibrated by defined standards prior to each measurement. The obtained data have been processed in the same time interval by the discharge/water level/precipitation vs. time diagram. Afterwards, the data were treated by the correlation analysis, for more accurate and more detailed comprehensive analysis.

## 3. Results

### 3.1. Meteorological Data

**Figure 3:**
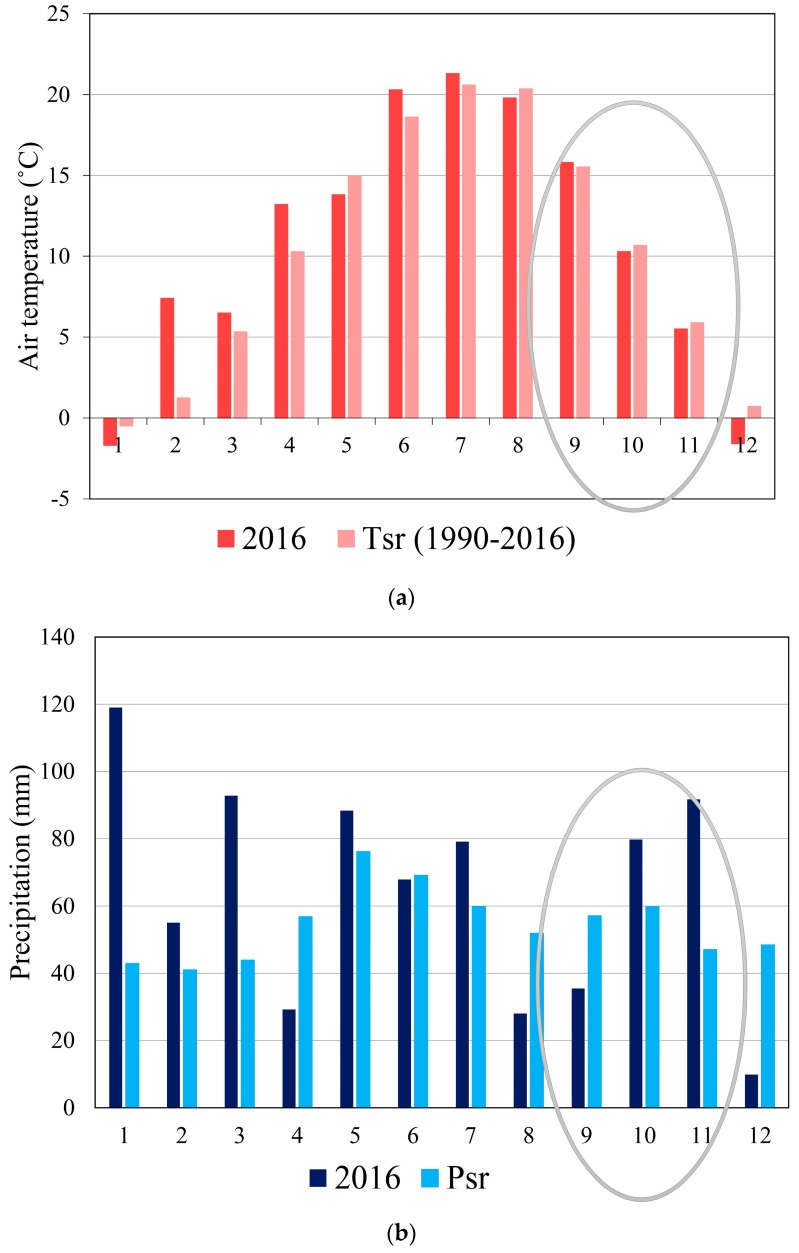
(**a**) Comparative diagram of the intra-annual distribution of air temperatures during 2016 and the multi-year period (1990–2016)—meteorological station Dimitrovgrad. (**b**) Comparative diagram of the intra-annual distribution of precipitation during 2016 and the multi-year period (1990–2016)—meteorological station Dimitrovgrad. The data enclosed in ovals in both panels were obtained during the three-month period (September, October, and November) when the survey was conducted.

The research was conducted over a three-month period in 2016, from early September to late November, and the meteorological characteristics of the area in this period are presented in Figure 3.

The measured precipitation values were lower in September and higher in October and November compared to the long-term average precipitation sums. The most significant deviations in total precipitation sums were recorded in November 2016 compared to the considered long-term level, with precipitation in 2016 nearly doubling compared to the long-term period. In September, there was a complete absence of precipitation for a period of 20 days, while the period without precipitation in October lasted for 15 days. The results of daily temperature and precipitation values for the considered three-month period were used in the analysis of the obtained hydrological and hydrogeological research results to define the degree of impact on the hydrogeological environment.

### 3.2. Discharge Regimes

The discharge of thermal waters was detected in the riverbed and in its immediate catchment area.

**Figure 4:**
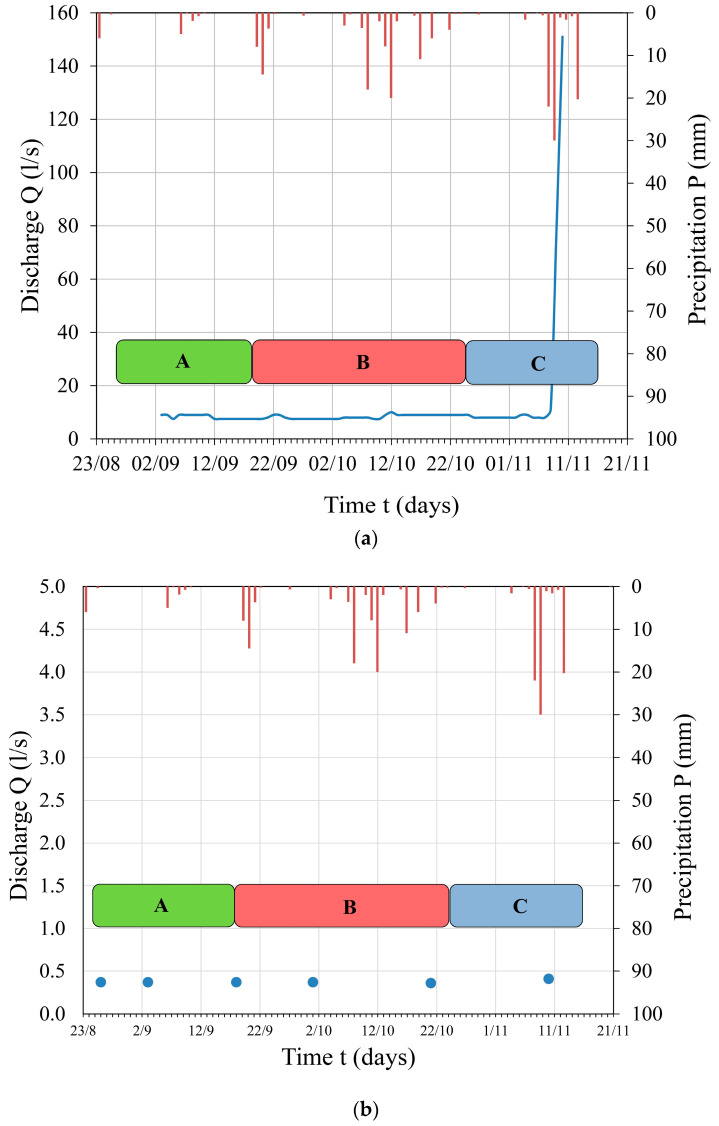
(**a**) Hydrograph of the *Izvor* spring depending on precipitation through different tunnel operation regimes: A, B, and C; (**b**) Hydrograph of the *Berilovac* spring depending on precipitation through different tunnel operation regimes: A, B, and C (given in different colors as explained previously, see Table 1). Red lines in figure are the precipitation values, while the blue dots are the discharge values.

Only at the Izvor spring was discharge measured using the hydrological method, where data on water level were collected, and discharge values were obtained through hydrological measurements. The discharge values measured at this karst spring are depicted by a hydrograph in Figure 4a. The results of the measured discharge at the Berilovac spring (volumetric method) are presented in the form of a hydrograph, with comparative values of daily precipitation sums, as shown in Figure 4b.

**Figure 5:**
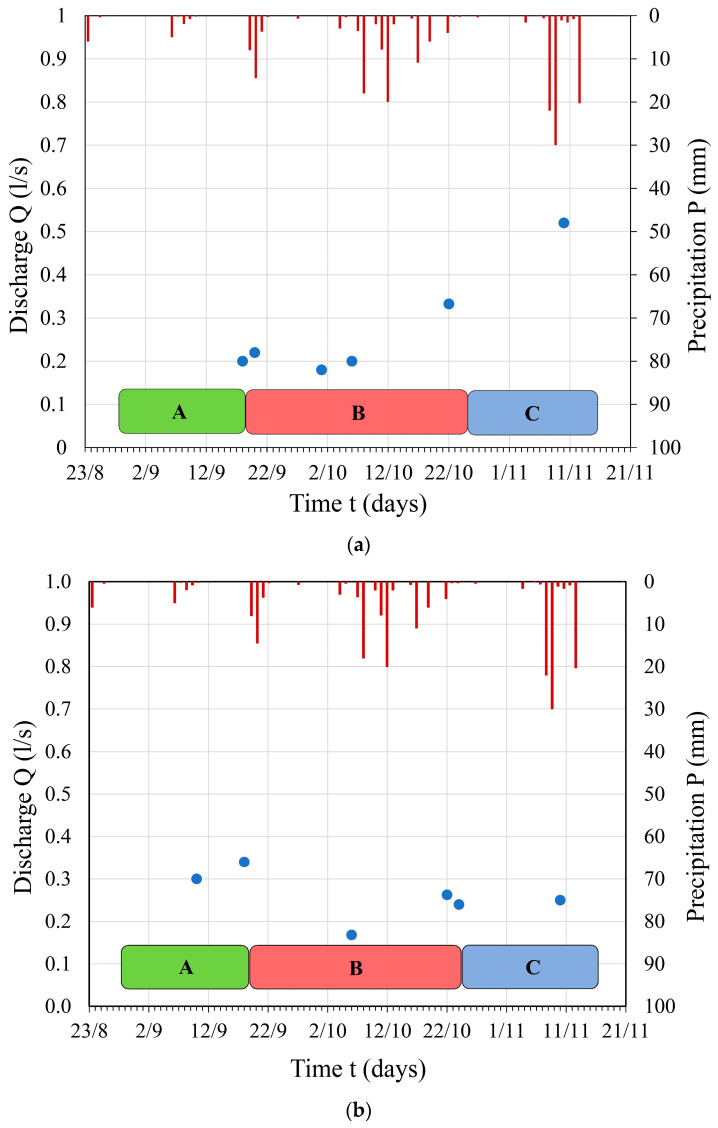
(**a**) Hydrograph of the Nišor 2 spring depending on precipitation through different tunnel operation regimes: A, B, and C; (**b**) Hydrograph of the Dobri Do spring depending on precipitation through different tunnel operation regimes: A, B, and C. Red lines in figure are the precipitation values, while the blue dots are the discharge values.

Measurements of discharge at the Nišor 1 and 2 springs were conducted using the volumetric method, and the results of the measured discharge at the Nišor 2 spring are presented in a hydrograph, with comparative values of daily precipitation sums shown in Figure 5a. The results of discharge at the Nišor 1 spring are not presented due to the small quantities measured, ranging from 0.006 L/s to 0.007 L/s, throughout the entire observation period. The results of discharge at the Dobri Do spring are depicted in a hydrograph in Figure 5b.

The discharge of the Izvor spring remained quite uniform throughout the entire observation period. During the research period, no changes caused by different tunnel operation regimes were recorded (during phases A, B, and C). The only change during the observation period was on 10 November 2016, but it was caused by heavy precipitation recorded from 8 November 2016 to 11 November 2016. The measured discharge values at the Glame spring remained constant throughout the entire observation period, at Q = 0.01 L/s. Similarly, at the Berilovac and Nišor 1–2, no significant changes in discharge were observed that could be attributed to the different operation regime of the artificial structure; instead, they were directly influenced by the pluviographic regime. The discharge of the Dobri Do spring measured on 10 June 2016, 18 days after the tunnel was emptied, was half as much (Q = 0.17 L/s) compared to the discharge measured on 10 September 2016 and 18 October 2016 (Q = 0.30 L/s and Q = 0.34 L/s), when the tunnel was in operation (phase A). On 22 October 2016 when the tunnel was still out of operation, there was an increase in discharge at the Dobri Do spring to Q = 0.26 L/s.

**Figure 6:**
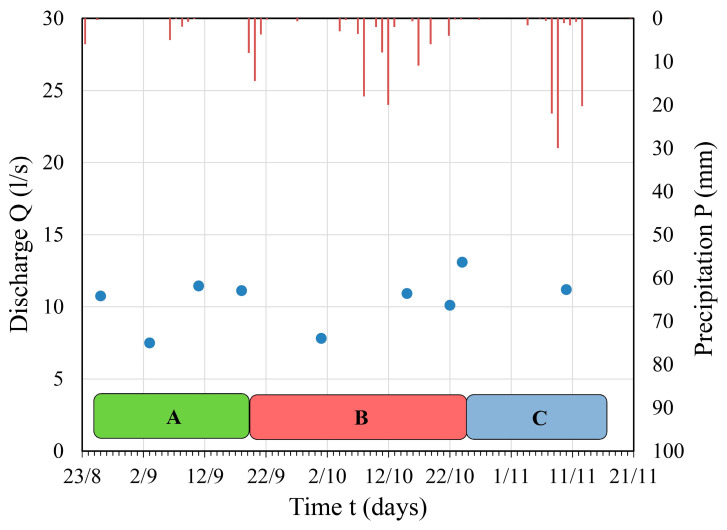
Hydrograph of the Dag Banjica spring depending on precipitation through different tunnel operation regimes (A, B, C).

At the thermal spring Dag Banjica (the main occurrence, or primitively tapped spring), discharge was measured, and the results are provided in Figure 6.

The discharge of the Dag Banjica thermal spring, enabled by the regional tectonics of the study area (Vidlička thrust), indicated a quite uniform discharge regime. Based on the discharge of this spring, it is observed that there is no direct dependence between precipitation and discharge. Dag Banjica, as the most significant phenomenon in this thermal spring discharge zone (primitive captured spring), has the highest temperature of 29.6 °C.

### 3.3. Physicochemical Properties of the Springs

**Figure 7:**
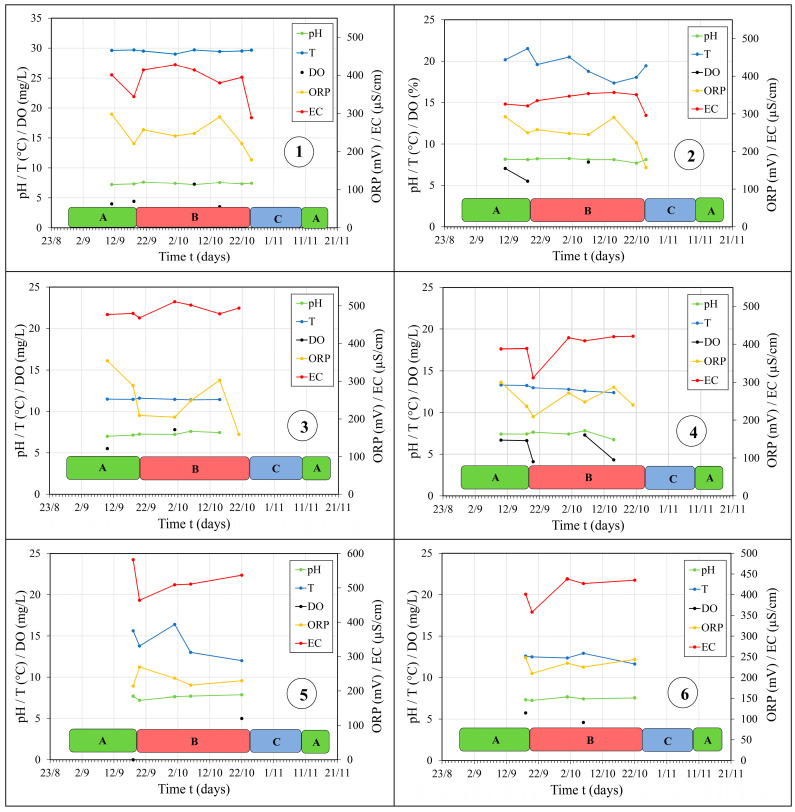

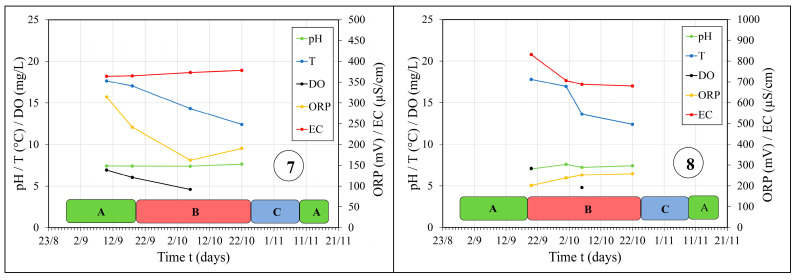
Physicochemical parameters of groundwater at the springs throughout the observation phases: ①—Dag Banjica, ②—Dobrodolska River, ③—Izvor, ④—Berilovac, ⑤—Nisor 1, ⑥—Nisor 2, ⑦—Dobri Do, ⑧—Glame. Different tunnel operation regimes labeled as A, B and C, and in different colors (as explained previously, Table 1).

Changes in all physicochemical parameters of the springs’ groundwater are depicted in Figure 7, while the chemical characteristics of the groundwater are listed in Table 5.

Physicochemical parameters of water in the Dobrodolska River observed in situ in this zone showed that during different tunnel operation regimes, observed through all phases (A, B, and C), the regime of physicochemical parameters was quite stable. The pH value was constant, while the water temperature was variable. Temperature fluctuations mainly occurred due to the mixing of thermal waters with constant temperatures and waters from cold springs, as well as surface water whose temperature was directly influenced by the atmospheric temperature regime. ORP and EC values were quite stable during different phases of the hydraulic tunnel operation regime, indicating that the qualitative regime of groundwater observed in the considered zone of the Dobrodolska River was not altered throughout the research period. The anionic and cationic composition of the sampled surface water from the Dobrodolska River (in the mixing zone with thermal waters) showed great similarity to the waters from the Dag Banjica spring (comparing station ① to station ②, Figure 7). These waters were predominantly of the Ca-Mg-HCO_3_ type, except during phase B when the water transitioned to the Ca-HCO_3_ type. Such a change in the chemical type of water is most likely due to the mixing of thermal waters with river water, which becomes dominant during periods of intense precipitation.

During all phases of different tunnel operation regimes, the chemical composition of water remained unchanged at the Izvor spring (Ca-HCO_3_ type) without any evident influence of transported/accumulated waters, while at the Berilovac spring during phase C, there was a change in the concentrations of Ca and Mg ions, which is solely due to the dominant process of limestone, or dolomitic, rock dissolution at a certain moment. Observation of the qualitative characteristics of groundwater at the Izvor and Berilovac springs, primarily the in situ measured physicochemical parameters, confirmed the assumption made based on the analysis of quantitative characteristics, that the different operation regime of the hydropower tunnel did not affect the change in the qualitative characteristics of the water from these springs. The ORP parameter value indicated that precipitation mostly affects the state of groundwater in the karst conduit that drains through this spring. Based on the anionic and cationic composition of groundwater, at these two springs, it is evident that they are typical limestone waters dominated by HCO_3_ and Ca ions.

The measured physicochemical parameters of groundwater at the Nišor 1 and 2 springs did not indicate significant changes. The only significant changes in the considered parameters were recorded regarding the change in the temperature regime of these waters. The temperature of groundwater fluctuated depending on the air temperature, which is especially pronounced at the Nišor 1 spring. At the Nišor 2 spring, temperature oscillations were milder but also caused by air temperature changes. The change in the anionic and cationic composition of groundwater from both considered springs indicates that they are Ca-HCO_3_ type waters, which did not change during the different phases of the tunnel operation regime.

Physicochemical parameters measured in the field at the Glame spring indicated only a change in the temperature regime, which is directly dependent on the air temperature. The groundwater flowing from the Glame spring is of the Ca-HCO_3_-SO_4_ type, with detected changes in Mg, Na+K, and SO_4_ ions during the different phases of the tunnel operation regime. During phase B, precipitation (three series of samples) in the groundwater from Glame spring was recorded, which probably caused an increase in Na+K ions that can reach the spring due to leaching of the surface area/recharge zone where animal fertilizers (manure) are present. Leaching of manure and infiltration of such waters can lead to an increase in the Na ion content in groundwater [34]. The increase in SO_4_ ion content is most likely due to a rain episode recorded during phase B in all considered springs formed in local hydrogeological systems. In rural areas with untouched forests, it has been found that rainwater has the most prevalent SO_4_ ion [40].

The results of the physicochemical parameters of groundwater at the Dobri Do spring showed that the most significant changes occurred in the temperature regime of the groundwater. During phase B, when the tunnel was out of operation, in addition to the recorded decrease in discharge at this spring, during the same period/series of measurements, there was a decrease in the groundwater temperature by 3 °C, while the ORP value decreased by 80 mV.

All observed physicochemical parameters of sampled waters from Dag Banjica thermal spring were constant throughout the entire observation period. Based on the anionic and cationic composition, the water of the Dag Banjica thermal spring is primarily of the Ca-Mg-HCO_3_ type, and the type of water did not change during the different observation phases. Compared to all other considered springs, this spring has the most constant concentrations of all components of the chemical composition of groundwater.

### 3.4. Hydrometric Station Assessment

**Figure 8:**
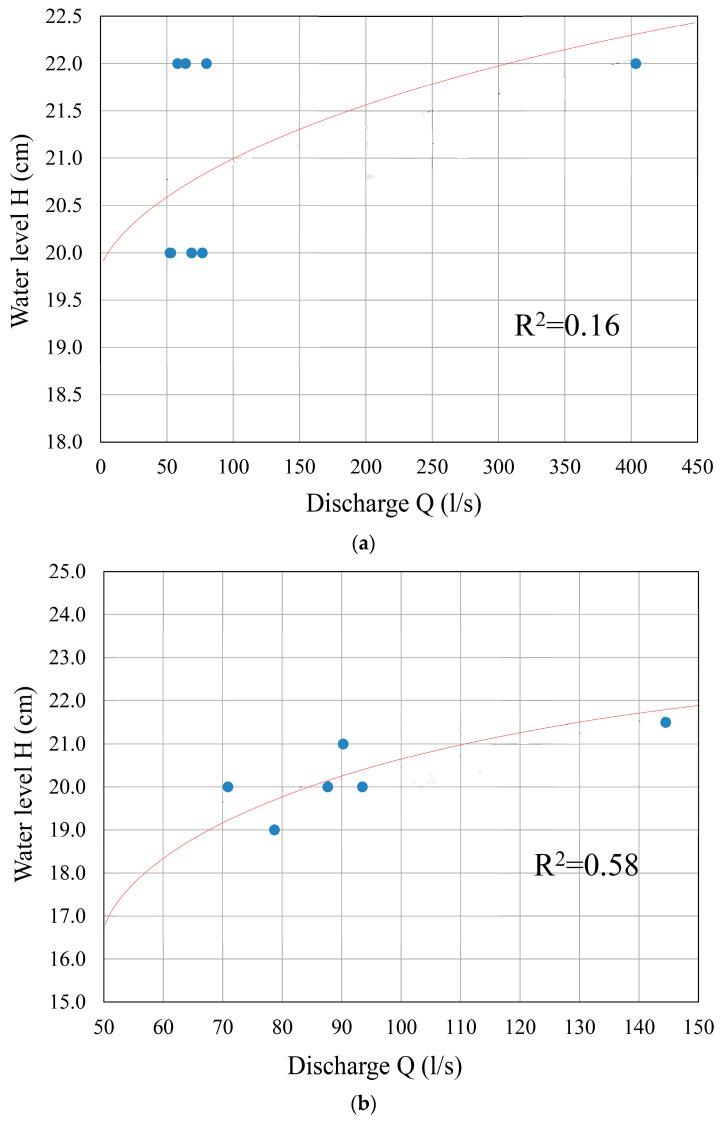
Determination coefficient, R^2^, for water level (H) and discharge (Q) on (**a**) gauge 1 and (**b**) gauge 2 (stations ① and ②, respectively). The regression fits the H-Q equation, H = $\sqrt{aQ}-b$, in the gauges; however, the determination coefficient in gauge 1 being smaller than in gauge 2 indicates a weaker correlation, mainly due to the presence of thermal springs in analyzed zone, not dependent on inflow from springs charged directly by precipitation.

Observations of the Dobrodolska River regime at the first hydrometric station, which was installed in the zone where the Dobrodolska River enters the area of thermal groundwater discharge, indicated a constant inflow of water from the Cretaceous limestones without detected significant changes in discharge and water levels observed through periods of different operation regimes of the hydraulic tunnel. The hydrograph of the Dobrodolska River (Figure 8a) shows that the discharge varied in the range of 50 to 80 L/s during almost the entire observation period, without a specific pattern indicating that changes in the river regime were caused by different tunnel operation regimes. In conditions of the re-established tunnel operation regime (17 days after tunnel charging), the highest recorded discharge at this station was 403.5 L/s. Such a significant increase in Dobrodolska River discharge in the considered station zone is primarily due to the pluviographic regime, i.e., intense precipitation that occurred during the period from 8 October 2016 to 11 November 2016, when a cumulative precipitation of 52.6 mm was recorded. This regime is also typical for surface streams formed on karst terrains, where rainy periods lead to groundwater/karst conduit filling.

At gauge 2 (Figure 8b), the water level remained almost constant throughout the observation period, with values around 20 cm. The only significant deviation in water level was recorded from 15 September 2016 to 21 September 2016, when the water level was 21 cm. The increase in water level at station 2 of 1 cm is most likely due to the unstable regime of thermal spring discharge, as indicated by the discharge measurements at the Dag Banjica thermal spring, as well as the specific geological and hydrogeological conditions of the entire thermal zone. However, despite the observed minor oscillatory changes in the surface stream level/water level, there were no significant discharge fluctuations (up to ±20 L/s) from 2 September 2016 to 15 October 2016. On 22 October 2016 and 24 October 2016, a significant discharge oscillation of ±60 L/s was recorded for the Dobrodolska River at this gauge, which is also a consequence of the Dag Banjica spring discharge regime and probably the discharge of all thermal springs formed in the Vidlička Nappe zone. This is supported by the fact that the highest discharge at the Dag Banjica spring (station 2, Figure 8b) was recorded precisely at these moments. The dependence of discharge and water level at this gauge shows a better correlation than at gauge 1 (Figure 8a), indicating the existence of a more stable discharge regime. It is confirmed by the values of the coefficient of determination (R^2^) for the two variables (in this case, water level and discharge) in Figure 8a (0.16) and Figure 8b (0.58): the R^2^ value in gauge 1 is smaller than in gauge 2, meaning the weaker correlation is in the former one, based on Chaddock’s scale [41] for the interpretation of correlation analysis (Table 6). This situation is primarily due to the presence of thermal springs in the considered zone and their constant discharge, which is not dependent on inflow from springs formed in local hydrogeological systems (directly dependent on precipitation) but rather on constant inflow of groundwater formed in deep, regional hydrogeological systems.

### 3.5. Impact of Artificial Structure on Hydrogeological Environment

**Figure 9:**
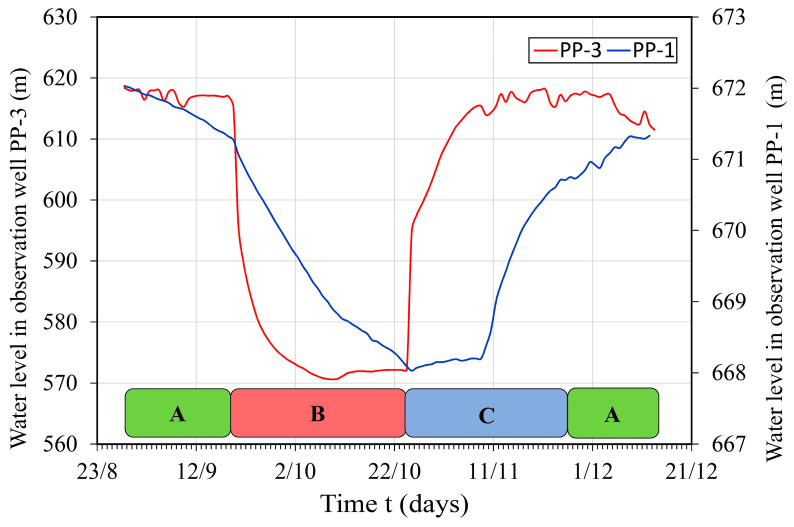
Comparative diagram of changes in groundwater levels at piezometers PP-1 and PP-3 (observation interval of 6 h) through different tunnel operation regimes: (A, B, C) [10].

Oscillations in groundwater levels at piezometer PP-1, which is the closest location to the reservoir (corresponding to tunnel station 1 + 400 m), and piezometer PP-3, which is installed in highly karstified limestones (ages K_1_^3^ and K_1_^3+4^) positioned in the Dobrodolska syncline, at tunnel station 4 + 200 m, are shown in a comparative diagram in Figure 9 [10]. The visible impact of the artificial structure on the natural hydrogeological environment was recorded at piezometer PP-1, while drastic changes in the oscillations of groundwater levels were recorded at piezometer PP-3, ranging from ±42 m [35]. The physicochemical parameters of groundwater in piezometers PP-1 and PP-3 showed drastic changes during different phases of the operation of the artificial structure, especially the EC parameter [10,35], as well as changes in the anionic and cationic composition [10].

### 3.6. Cross-Correlation Analyses

**Figure 10:**
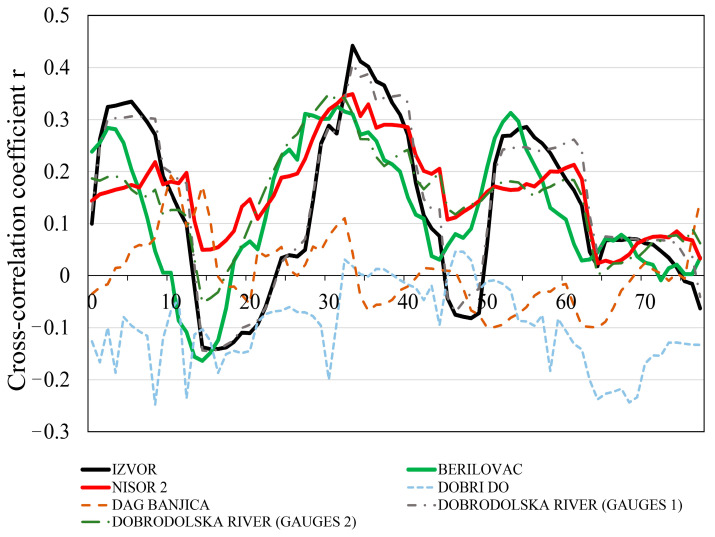
Correlation coefficient between daily precipitation and daily discharge converted from water levels measured at five springs of Izvor, Berilovac, Nišor 2, Dobri Do, Dag Banjica and two measurement stations on the Dobrodolska River (gauges 1 and 2) using flow rate function (Q = f(H)) at each spring and gauge.

The cross-correlation analysis between the daily precipitation on the daily discharges of five springs (Izvor, Berilovac, Nišor 2, Dobri Do, and Dag Banjica) and two measuring stations (gauges 1 and 2) on the Dobrodolska River for the entire observation period shows good correlations between the daily precipitation and the daily discharge at the Izvor, Nišor 2, and Berilovac springs (Figure 10). The cross-correlation coefficients for these three springs (0.442, 0.348, and 0.325, respectively) indicate the propagation of precipitation occurring within 2 to 4 days. The good correlation between the precipitation regime and the discharge regime of the Dobrodolska River at the two measurement stations (0.407 and 0.349) with a time lag of 3 to 4 days follows the previous three springs, which is expected since the Dobrodolska River is largely fed by karst springs.

The weakest correlation between these two variables (max 0.046) was established at the Dobri Do spring, which further indicates the unreliability of the data measured at this spring. The correlation coefficient in the case of the thermal spring Dag Banjica indicates a weak correlation between the discharge regime and the precipitation regime, with a long time lag, which is certainly a result of deep circulation and the specific discharge regime of this spring.

## 4. Discussion

The tunnel’s impact on the hydrogeological environment has been proven at the closest positions by measuring in PP-3 piezometer, but also in PP-1. Our recent work on this topic [42] proved a strong correlation between real groundwater-level data in piezometer PP-3 and simulated data at the outlet channel, with a coefficient of determination of R^2^ = 0.96, meaning a very strong correlation on Chaddock’s scale [41]. The equation of regression describes the way the dependent variable is related to the one or more independent variables. Although the hydrograph at the Dobri Do spring does not indicate any drastic changes during the observation period, one significant change was observed during phase B (tunnel out of operation) in Figure 5b, which may indicate a potential influence of the tunnel on the discharge regime of this spring. However, this change should be taken with caution considering that the obtained results were not observed at the main discharge zone of this spring. The increase in the discharge at Dobri Do on 22 October 2016 is in contrary to what would be expected if there was an anthropogenic influence, and most likely indicates that the catchment area of the spring is directly influenced by atmospheric precipitation (rain) caused by the recorded precipitation on 17 October 2016, which drained into the catchment area five days earlier (10.9 mm). The refilling of the tunnel did not lead to a significant increase in discharge.

The changes in the temperature and redox regime of the groundwater in Dobri Do spring can be due to the decline in groundwater levels due to the release of pressure exerted by the hydraulic tunnel on the hydrogeological environment. However, the decline in groundwater levels in this karst conduit may be due to the absence of precipitation and the onset of a recession period. Since this spring is in close proximity to piezometer PP-3 and is located in an extremely karstified area, there is rapid propagation of precipitation into the karst conduit. The precipitation that occurred at the end of phase B sampling prevented a clear understanding of the potential impact of the pressure tunnel regime on the discharge regime of the Dobri Do spring. The poor correlation between the precipitation and discharge indicates that the data from this spring are not reliable, primarily due to the fact that the observations were not conducted at the main discharge zone of the spring.

The uniform discharge regime of Dag Banjica and the lack of dependence between precipitation and discharge is another confirmation that the water flowing in the entire zone of this spring, including the spring itself, comes from significant depths, where the recharge zone is quite distant from the discharge zone. Thus, deep groundwater circulation does not have direct contact with waters transported by the hydraulic tunnel. The qualitative characteristics of the waters from the Dag Banjica thermal spring indicate that the chemical composition did not change due to atmospheric precipitation, which is a result of the much more distant recharge zone compared to the discharge zone, i.e., the existence of deep/regional groundwater circulation. This is confirmed by the cross-correlation diagram and the cross-correlation coefficient of the two variables (precipitation and discharge).

The extensive collection and analysis of data throughout the research process have enabled the understanding of natural conditions of the geological/hydrogeological environment as well as the conditions resulting from anthropogenic influence, i.e., the operation of the hydraulic tunnel. Raposo et al. [28] detected a tunnel impact in fractured granitic bedrock in northwestern Spain, causing significant damage to private groundwater users, which gives rise to making decisions about rectification measures, and is comparable to our results. A confirmation and quantification of tunnel impact often implies the need for redirection and redesign in tunnel constructions, particularly in karst areas (such as the Mediterranean basin [28,30,33], Alps [32], or large areas of China [21,43]), being significantly aligned with the results of this study. It has also been shown that various other man-made structures (such as basements of buildings, deep foundations, dams, etc.) produce a significant impact on the groundwater regime in urban aquifers, either as an obstacle to the flow or through the disturbance of the groundwater budget of the flow system [43,44].

There is an evident connection between the two variables—in this case precipitation and spring discharge, and the Dobrodolska River flow rate (both gauges)—meaning that the degree of the connection is a correlation. The correlation analysis defines how the independent variable (precipitation) influences the dependent variable (discharge), and the strongest correlations were found at Izvor, Nišor 2, and Berilovac. Precipitation exerts the dominant impact on the spring discharge regime of these three springs. Although during the conducted hydrological research on the Dobrodolska River it was noted that the number of collected and analyzed data on water levels and discharge were insufficient to define its natural regime, the measured discharges proved to be extremely important in resolving the issue and determining the potential impact of the tunnel operation on the analyzed watercourse, despite the research being conducted during a hydrologically unfavorable period, i.e., the rainy season. This is confirmed by the correlation between precipitation and discharge at both measurement stations. Certainly, successive hydrological observations on the Dobrodolska River did not indicate that the detected changes could be attributed to different operation regimes of the hydraulic tunnel. Potentially, both the spatial and, above all, the altitude position of the measurement stations and the observed zone on the Dobrodolska River, ranging from elevations of 471 to 432 m.a.s.l., indicate that the tunnel, which operated at a pressure of about 4 bars, is situated above the considered zone. However, the pressure resulting from the transport of accumulated tunnel water and its impact on the groundwater must not be considered only from the perspective of the vertical direction but must be analyzed equally in all directions. The quantitative analysis of the Izvor and Berilovac springs indicated that the measured changes were caused by changes in hydrometeorological conditions, i.e., the pluviographic regime, which was also confirmed by the cross-correlation coefficients. Based on the elevations (terrain elevations) at which these springs are located (409 m.a.s.l. and 406 m.a.s.l.) relative to the mean elevation of the tunnel (552 m.a.s.l.), it can be noted that they are below the potential influence zone of tunnel operation, but only if it is considered that the tunnel’s influence exists in zones above its mean elevation, which is not entirely accurate. The quantitative characteristics of the Nišor 1 and 2 springs, observed through different phases of the operation regime of the hydraulic tunnel, did not significantly change. The constant discharge from the Nišor 1 spring, and the fact that it is located at an elevation of 776 m.a.s.l., well above the mean elevation of the tunnel (552 m.a.s.l.), and above the elevation of the measured pressure (approximately 4 bars/41 mH_2_O) of transported tunnel water (the elevation of the upper water in the reservoir was 606 m), and the visible temperature fluctuations caused by air temperature fluctuations, indicate that this spring belongs to a local hydrogeological system formed in a relatively shallow zone, with closely dependent recharge and discharge areas directly influenced by atmospheric conditions. The Nišor 2 spring is located at a much higher elevation compared to the operational pressure elevation of the tunnel (130 m above the operational pressure elevation), far above the potential influence of different operation regimes of the pressurized tunnel, as indicated by the results of discharge measurements.

The elevation of the Glama spring (terrain elevation 691 m.a.s.l., or 139 m above the mean elevation of the tunnel, or 85 m above the achieved operational pressure of the tunnel at a reservoir elevation of 606 m) indicates that transported tunnel water does not affect changes in its discharge regime or quantitative characteristics. The maximum operational pressure in the tunnel is achieved when raising the water column by 81 m, or about 8 bars, which is an additional confirmation that this spring, even under extreme anthropogenic conditions, is not located in the influence zone of tunnel operation.

The results of the quantitative characteristics of the water sampled at the Dobri Do spring and their analysis must be approached with caution because the observations were not carried out in the direct discharge zone of the spring but at a fountain located in the village of the same name and at an elevation of 623 m above sea level (m.a.s.l.), which did not determine the elevation of the terrain where the direct discharge of the spring occurs. Although the analyzed fountain is very close to the observation piezometer PP-3, where the most drastic fluctuations in groundwater levels were recorded during the research period, a comparative analysis and direct dependence between these two observation points are entirely unfounded since the main discharge zone of the Dobri Do spring is distant from the analyzed fountain. However, the fact that research at piezometer PP-3 indicated significant changes in the water column in the hydrogeological environment (of 43 m) during different phases of the operation regime of the hydraulic tunnel suggests that changes in discharge at this spring could be an indicator that the mentioned piezometer and the Dobri Do spring are within the same karstic conduit, which is directly influenced by the operation of the tunnel. At this spring, besides noticeable changes in Mg and SO_4_ ions during phase B when the tunnel was out of operation, caused by precipitation, significant changes in Na+K ions were also recorded, most likely resulting from the increased content of Na ions, which can enter groundwater as a product of anthropogenic activity [34]. Elevated Na ion content was also detected in the waters of piezometer PP-3, which once again indicates a potentially shared karstic conduit that drains through this piezometer and the Dobri Do spring. The first assumption is that the hydraulic tunnel potentially affects the groundwater regime of this spring, and the change in groundwater levels occurred due to rain (as in the case of piezometer PP-3). The second assumption is that the hydraulic tunnel did not affect the groundwater levels, and the discharge from this spring is primarily due to a local system where discharge dynamics are exclusively dependent on prevailing recharge conditions at a given time. However, drawing specific conclusions regarding changes in the quantity and quality of the Dobri Do spring caused by different operation regimes of the tunnel would be entirely unfounded given the facts mentioned above. Therefore, observations at this spring indicated that far more detailed research is needed at this observation point than what was conducted during this research process.

The consistent discharge regime of the Dag Banjica thermal spring at an elevation of 469 m.a.s.l., or 137 m below the operational pressure of the tunnel (reservoir elevation 606 m), indicates that even the tunnel with a maximum operational pressure of an 81 m water column is not in the potential influence zone of the natural regime of this spring. However, the previously defined regional structural–geological characteristics of the study area and the formed Vidlička thrust in this zone, which allowed the discharge of water from deep/regional hydrogeological systems where the recharge zone is distant from the discharge zone, further indicate that different operation regimes of the tunnel and the pressure of transported accumulation waters do not affect the regime of these springs. This was also indicated by the collected data on the water quality sampled from this thermal spring, with a constant discharge temperature of 29.6 °C.

Based on the detailed monitoring conducted on the established network during the observation period, the detected changes at piezometers PP-1 and PP-3 [10,35] indicated a direct impact of the artificial structure on the natural hydrogeological environment, while such pronounced changes were not detected at the seven springs and one river analyzed.

In order to be able to thoroughly analyze changes in the discharge regime of the analyzed springs, a longer monitoring period should be arranged for at least a year of observation (and a more detailed monitoring network). Also, a longer period of the tunnel being out of operation is advisable for more profound proof of no interaction between the discharge regime of groundwater at natural springs and artificial structures. It has been proposed recently that the impact of a tunnel on karst areas manifests in gradual changes of soil properties (physical and chemical), soil erosion rate, plants’ growth rate, etc. [21]. However, the understanding of these processes and mechanisms is still poor, and further research of this topic is needed.

The influence between the hydraulic tunnel and hydrogeological environment is mutual. Therefore, it is needed to conduct a more detailed research of the tunnel impact, particularly in case of the increased change in tunnel structure in the study area, so that the analyzed results could be more meaningful. As the survey period of this study was short (three months), it is safe to assume that the impact of other structures on the hydrological environment during this period is constant. However, by monitoring over a long period and comparing the fluctuation patterns when new artificial structures are built, it should be possible to investigate the impact of artificial structures on the hydrological environment.

## 5. Conclusions

By conducting a detailed analysis of the quantitative and qualitative characteristics of groundwater from multiple springs, piezometers, river, and the hydraulic tunnel, the following conclusions can be drawn:

(1) It was possible to assess and forecast the risks of the impact of a hydraulic structure on the hydrogeological environment by detailed formation of a monitoring network and applying the appropriate research methods described in this study.
(2) The conducted research on the qualitative/quantitative regime of groundwater at the springs did not show a direct dependence on the tunnel, but these springs were crucial for defining the types of hydrogeological systems, especially those located well above or well below the potential influence of the hydraulic tunnel. Although the elevation of all analyzed springs initially may indicate the absence of a direct connection to the tunnel operation regime, water pressure must be considered not only in the vertical direction, but in all directions equally, as it being transmitted.
(3) The extremely dynamic connection and mixing of waters transported by the tunnel under pressure with groundwater (recorded in piezometer PP-3 along the tunnel route) indicate the direct impact of the artificial structure on the natural hydrogeological environment. On the other hand, changes in groundwater levels and chemical composition in the second piezometer PP-1, as well as in the hydraulic tunnel itself, indicated the direct impact of the tunnel on the directions of groundwater flow, which were altered due to the operation of the artificial structure.
(4) Hydrogeological structures were formed along the tunnel route, unlike the analyzed springs, which are located at a certain distance from the artificial structure. Therefore, the observation period of 108 days was sufficient to detect changes in the piezometers, but not long enough to detect changes in the discharge regime of the analyzed springs. It is recommended to establish a monitoring network for a longer observation (one year or more) with a longer period of the tunnel being out of operation, for further proof that the artificial structure does not affect the discharge regime of the groundwater at natural springs.

By integrating piezometer readings, hydrogeological investigations, and advanced physicochemical monitoring and analyses, we provide valuable insights applicable to similar hydrogeological systems globally. The findings of this study point out the importance of detailed assessments in areas nearby the hydropower tunnels to successfully mitigate risks. This approach not only improves the understanding of hydrogeological systems and groundwater dynamics, but can also predict and recommend efficient strategies for mitigating a detrimental impact on the environment in diverse geological settings. This study, therefore, provides a contribution in improving practices for hydrogeological management and planning, and promotes more sustainable development of hydropower systems worldwide.

## Figures and Tables

**Table 1:** Observation periods during which the research was conducted [10]. Three colors distinguish the three modes of tunnel’s operation in the study.

Phase	Tunnel Operation Regime	Beginning–End of the Observation Period	Total Duration of the Phase (Days)
A	Operating mode/pressurized tunnel/in operation	28 August 2016–19 September 2016	23 days
B	Tunnel out of operation	19 September 2016–24 October 2016	35 days
C	Tunnel back in operation/resumed operating mode	24 October 2016–10 November 2016 (13 December 2016)	50 days (83 days)

**Table 2:** Technical details of the EasyFlow instrument used in this study.

measurement range	From 0.1 to 99,900 L/s
precision	<5%
repetitivity	±1%
tracer type	NaCl
tracer amount	from 10 g to 100 kg of salt
ideal tracer mix	between 5 and 20 g of salt per L/s of assessed flow rate
charging	3 × 1.5 V alcaline batteries
durability	~100 h (at standard conditions)
connection	over link RS-232
device dimensions/weight	230 × 150 × 80 mm/620 g
water resistivity	IP65
salt concentration	
measurement range	0 to 3200 mg/L
sensitivity	1 mg/L
precision	<1%
temperature	
measurement range	0 to +40 °C
precision	±0.2 °C

**Table 3:** Technical characteristics of HANNA multiparameter probe HI9298194 used in study.

	pH	Temperature, °C	ORP, mV	DO, %	EC, mS/cm	TDS, g/L
measurement range	0.00–14.00	−5.00–55.00	±2000.0	0.0–500.0	0–200	0–400
resolution	0.001	0.01	0.1	0.1	0.001–400.0	0.001–400.0
accuracy	±0.02	±0.15	±1.0	±1.5–3.0	±1.0%	±1.0%
calibration	*	automatic (in one adapted point)				based on EC calibration

* automatic in 1, 2, or 3 points, with automatic recognition of 5 buffers (pH 4.01, 6.86, 7.01, 9.18, 10.01).

**Table 4:** Overview of chemical parameters and methods of their monitoring.

mg/L	Methods
HCO_3_^−^	volumetry
CO_3_^−^	volumetry
Cl^−^	potentiometry
SO_4_^2−^	turbidimetry
NO_3_^−^	spectrophotometry
Na^+^	AAS—atomic absorption spectrophotometry, flame technology, acetylene-air
K^+^	AAS—atomic absorption spectrophotometry, flame technology, acetylene-air
Ca^2+^	AAS—atomic absorption spectrophotometry, flame technology, acetylene-nitrous oxide
Mg^2+^	AAS—atomic absorption spectrophotometry, flame technology, acetylene-nitrous oxide
Fe_total_	AAS—atomic absorption spectrophotometry, flame technology, acetylene-air
Sr^2+^	AAS—atomic absorption spectrophotometry, flame technology, acetylene-nitrous oxide
Li^+^	AAS—atomic absorption spectrophotometry, flame technology, acetylene-air
H_2_S	gas volumetry

**Table 5:** Chemical type of groundwater at the springs throughout the observation phases.

Tunnel Operation Regime Phase	Name of the Spring	Type of Groundwater	Tunnel Operation Regime Phase	Name of the Spring	Type of Groundwater
A	Dobrodolska River	Ca-Mg-HCO_3_	A	Dag Banjica	Ca-Mg-HCO_3_
B		Ca-HCO_3_	B		Ca-Mg-HCO_3_
C		Ca-Mg-HCO_3_	C		Ca-Mg-HCO_3_
A	Izvor	Ca-HCO_3_	A	Berilovac	Ca-HCO_3_
B		Ca-HCO_3_	B		Ca-HCO_3_
C		Ca-HCO_3_	C		Ca-Mg-HCO_3_
A	Nisor 1	Ca-HCO_3_	A	Nisor 2	Ca-HCO_3_
B		Ca-HCO_3_	B		Ca-HCO_3_
C		Ca-HCO_3_	C		Ca-HCO_3_
A	Dobri Do	Ca-HCO_3_	A	Glame	Ca-HCO_3_-SO_4_
B		Ca-HCO_3_	B		Ca-HCO_3_-SO_4_
C		Ca-HCO_3_	C		Ca-HCO_3_-SO_4_

**Table 6:** Chaddock’s scale for interpretation of correlation analysis.

Coefficient of Determination	Interpretation
0.00	no correlation
0.00–0.25	weak correlation
0.25–0.64	moderate correlation
0.64–1.00	strong correlation
1.00	total correlation

## Data Availability

The data supporting reported results can be found at Mrs. Marina Čokorilo Ilić (first author).
